# Consequences of COVID-19 regulations on the competences of medical graduates and FM/GP interns – teachers' views

**DOI:** 10.1080/13814788.2022.2030589

**Published:** 2022-01-31

**Authors:** Manfred Maier

**Affiliations:** Department of General Practice, Centre for Public Health, Medical University of Vienna, Vienna, Austria


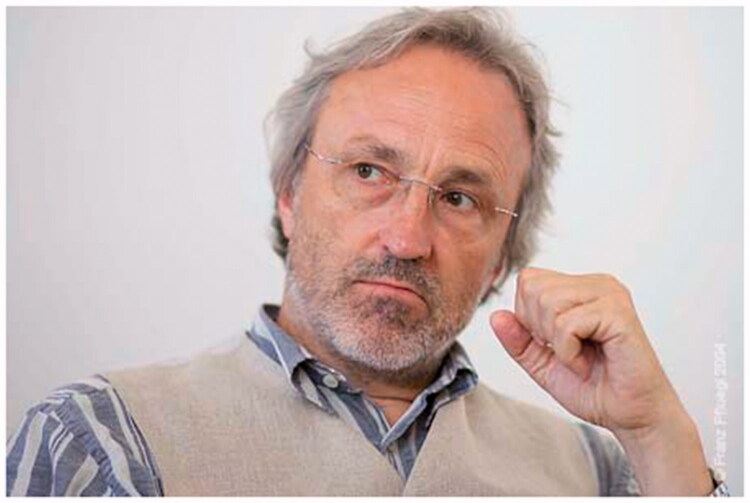


## Background

To reduce transmission of the coronavirus and to control the spread of COVID-19, several measures were introduced globally in early 2020. Among them was the sudden closure of educational institutions, including universities. In most countries, medical students were faced with suspension of clinical attachments and a significant change in their curriculum: theoretical lectures were presented online and clinical practice was temporarily cancelled, postponed and shortened.

After almost two years, these or similar measures are still in place, albeit with local variations. Overall, medical universities and medical educators seem to have responded quickly and creatively to the new educational challenges [1]. However, can medical students under these circumstances gain the skills, experiences and attitudes they require to become competent doctors [2]?

This question is, in particular, relevant for students who aim to work in General Practice/Family Medicine. Next to a broad knowledge base, the competences for this discipline require the ability to communicate empathically and efficiently with all kinds of patients. However, already before implementing the Covid 19- regulations, patients were frequently complaining about the lack of good communication with their doctors. Furthermore, students need to acquire clinical reasoning skills for the outpatient setting, away from the sheltered workplace in the hospital.

I did a short survey among colleagues and friends around the globe – Australia, Austria, Brazil, Estonia, Greece, Netherlands, Slovenia, Spain, Switzerland, Turkey and the UK – and asked for their views. In summary, there was agreement that the restrictions on teaching and training imposed both challenges and opportunities to Medical Faculties and Universities, that the organisation of new teaching formats and schedules was complex and that some students and teachers adapted better than others.

## Opportunities

Better use of technology for online learning is considered as a positive development. Some universities developed virtual workshops and other e-learning options such as the ‘flip the classroom concept’ for individual and group learning. These relatively new teaching formats seem to attract more participants. However, active participation and interactive discussion or dialogue is reduced. Control of attendance is obviously difficult, as are meaningful examinations in the new formats.

In some countries, students were involved in testing and vaccination programmes and some were able to help in homes for the elderly. Students gained hands-on experience working in a health care system challenged by a pandemic and learned how this affects their personal lives, and that of patients and their families. Consequently, students should have gained an increased understanding of the importance of Public Health and of epidemiology, primary care and prevention of infections.

In Slovenia, the Medical Faculty provided expertise to the whole University concerning how to adapt to the ever-changing epidemic. Moreover, at the beginning of the epidemic, where there was much uncertainty, medical students – supervised by their teachers – organised a call centre giving answers to questions from the public. The president of Slovenia recently awarded this initiative and service.

## Challenges

On the other hand, negative consequences are seen due to the restrictions on classical teaching formats and on practice-based training of current state of the art curricula. Due to reduced opportunities for clinical placements, especially to rural and remote areas and to foreign Universities as exchange students, students see fewer patients in person and bedside teaching and face-to-face learning is reduced. Isolation of infected people, the need for personal protective equipment and the fear of infection affects the training in essential skills such as palpation of patients.

Other negative consequences include the neglect of chronic diseases and non – communicable diseases or the diminished opportunity for interaction between students and teachers, which results in less discussion of presentation, condition or prognosis of patients with colleagues and teachers. The training of clinical reasoning often takes place without patients and their families.

There is less opportunity to practice communication skills and learn how and what patients tell the doctor in their native language or dialect. Although some universities have developed methods to adapt to an online format – for example, role-play – this works only for verbal and not for non-verbal communication. In some countries, vaccine hesitancy among health professionals and citizens has become a major challenge for health policy and society; however, students and trainees seem to be unprepared to discuss and argue with this special group of people.

The defined aims of modern medical curricula such as training in patient-centred care, integrated and team-based learning or interprofessional education are currently hard to accomplish. Many of my expert-colleagues see a deterioration of a meaningful medical education to a theory-based form of distance learning. This new reality for medical education and training suffers from an enormous lack of practical teaching and clinical subjects. This view is supported by a study from Turkey [3] where 90% of residents complained that the restrictions negatively affected the structure and content of their residency training. On a personal side, medical students reported social isolation, especially if they study far from home and family.

Students adjust to learn for new forms of examinations and accumulate theoretical knowledge without knowing how to implement it. A GP-colleague from the UK fears that young doctors might have become used to taking the dangerous shortcut directly from history to diagnostic investigation or even treatment.

How will these changes impact the thinking, clinical skills and attitudes of this generation of medical doctors, particularly of future GPs/FDs?

## Lessons to learn

The pandemic will not disappear in a few months and Medical Universities should, therefore, learn from the experiences so far. For both students and teachers training the skills helpful for virtual consultations seems to be a must. Graduates should be equipped with proper scientific training and thinking and be experienced in ethical discussions to successfully and empathically argue with patients or colleagues about vaccine hesitancy. Further, graduates should be strongly supported to manage their own well-being and development and training in difficult situations.
